# Prevalence of psychological symptoms among Ebola survivors and healthcare workers during the 2014-2015 Ebola outbreak in Sierra Leone: a cross-sectional study

**DOI:** 10.18632/oncotarget.14498

**Published:** 2017-01-10

**Authors:** Dong Ji, Ying-Jie Ji, Xue-Zhang Duan, Wen-Gang Li, Zhi-Qiang Sun, Xue-Ai Song, Yu-Hua Meng, Hong-Mei Tang, Fang Chu, Xiao-Xia Niu, Guo-Feng Chen, Jin Li, Hui-Juan Duan

**Affiliations:** ^1^ 302 Military Hospital of China, Beijing, China; ^2^ Sierra Leone-China Friendship Hospital, Freetown, Sierra Leone

**Keywords:** ebola virus disease, psychological symptoms, SCL-90-R, emerging infectious disease, emergency response plan

## Abstract

The 2014–2015 Ebola epidemic was considered to be the largest and most complex outbreak, which caused 11,310 reported deaths. The epidemic disease can cause a mental health crisis, however, there is only a small amount of scientific literature available related to this health issue so far. We evaluated the psychological symptoms of 161 participants including Ebola survivors and healthcare workers in Sierra Leone, analyzed the impact of job classification, education level on psychological status. We found that the order of total general severity index (GSI) scores from high to low was EVD survivors, SL medical staff, SL logistic staff, SL medical students, and Chinese medical staff. There were 5 dimensions (obsession-compulsion, anxiety, hostility, phobic anxiety, and paranoid ideation) extremely high in EVD survivors. GSI were associated with university education negatively. We believed our information is necessary to develop the comprehensive emergency response plan for emerging infectious disease outbreak.

## INTRODUCTION

Ebola virus disease (EVD) is a rare and fatal disease caused by one of the Ebola virus strains. It was spread by close contact with patients and by use of contaminated needles and syringes in hospitals/clinics[1–4]. The EVD outbreak in West Africa caused widespread public attention from 2014 to 2015. So the World Health Organization (WHO) urgently appealed to the international community to provide medical aid on 8 August 2014 [5]. In response to the requests of WHO and governments of the West Africa, Chinese government have deployed Chinese aid medical teams to the most affected Liberia and Sierra Leone to provide clinical care, infection control, health promotion and post-disaster reconstruction. The Chinese aid medical teams played a vital role for the success of this battle by quick response, professional EVD management and strong support to the local health system [6–8].

It had aroused the public`s fear and panic because the media initially focused too much on the high infectivity and case fatality. For the control of EVD, it is necessary to use the personal protective equipment (PPE), quarantine, and isolate in the process of infection control and prevention, which may be associated with further fear and anxiety [9, 10]. This general public response to new emerging infectious disease (EID) with extremely high morbidity and mortality is the distancing-blame-stigma pattern, which comprises distancing oneself from the EID, blame of persons for the disease's origin and spread, and stigmatization of EID patients [11]. Mental status of EVD survivors might have important health implication for EVD control, however, researchers mainly focused on clinical the manifestations and epidemiology of EVD, and almost neglected the study on psychosocial impact or distress associated with EVD [12].

Even staffs working in formal hospitals must undergo intensive protection training before they can deal with patients with high infectivity, it cannot be guaranteed that the medical staffs with advanced PPE according to the standard operation procedure are definitely safe. In the West African outbreak, healthcare workers had to cope with the deaths of colleagues, threats to their lives, and working excessive hours in addition to their own anxiety and fear of contamination. Furthermore, prolonged shift times might reduce work efficiency and increase the accidents risk. The psychological status of the healthcare workers treating Ebola patients is unclear and should be further evaluated [13].

Therefore, we employed the Symptoms Checklist 90-items, Revised (SCL-90-R), a most widely used questionnaire, to measure psychological symptoms of survivors, and healthcare workers not only before but also after contacting EVD, which will provide the direct and detailed data for dealing with furthermore regional or global public emergency. We hope that government pays more attention to psychological supports for survivors of EVD and gives consideration to emotional challenges for medical personnel who dispose with EVD.

## RESULTS

### Demographic information

The clinicians of the Chinese aid medical team treated EVD patients in an Ebola Treatment Center (ETC) in Jui Government Hospital, Sierra Leone. Being one of the best hospitals in Freetown, Jui Government Hospital received 773 EVD-suspected patients during the period of October 1 2014 and March 21, 2015, of whom 285 were confirmed to be infected with Ebola virus, 146 survived, and 139 died. The death rate of our ETC was 47.7%.

The total sample consisted of 161 participants and were divided into 5 categories according to working property, they were Sierra Leone (SL) medical staff (*n* = 59), SL logistic staff (*n* = 21), SL medical students (*n* = 22), and Chinese medical staff (*n* = 41), the other group consisted of 18 EVD survivors. The median age was 32.0 years (range, 12–80) and 81 (50.3%) were male. About two-thirds (63.4%) of the participants had university education. There were no significant differences on gender proportion among these 5 groups (*P* > 0.05), and the percentages of university education were increased gradually from 0 (EVD survivors) to 95.1% (Chinese medical staff) (Table 1).

**Table 1:** Chinical characteristics of healthcare workers and EVD survivors

Characteristics	EVD survivors (*n* = 18)	SL medical staff (*n* = 59)	SL Logistic staff (*n* = 21)	SL medical students (*n* = 22)	Chinese medical staff (*n* = 41)
Male, n (%)	9 (50.0)	29 (49.2)	10 (47.6)	12 (54.5)	21 (51.2)
Age, median (range), yrs	29 (12–80)	31 (24–43)	31 (27–41)	25 (23–31)	38 (25–0)
12–30 yrs, *n*(%)	8 (44.4)	33 (55.9)	10 (47.6)	20 (90.9)	4 (9.8)
31–50 yrs, *n*(%)	8 (44.4)	26 (44.1)	12 (52.4)	2 (9.1)	37 (90.2)
> 51 yrs, *n* (%)	2 (11.2)	0 (0)	0 (0)	0 (0)	0 (0)
Education					
College or below, *n* (%)	18 (100)	39 (66.1)	11 (52.4)	5 (22.7)	2 (4.9)
University or professional training, *n* (%)	0 (0)	20 (33.9)	10 (46.7)	17 (77.3)	39 (95.1)

### Psychological dimensions

**Figure 1:**
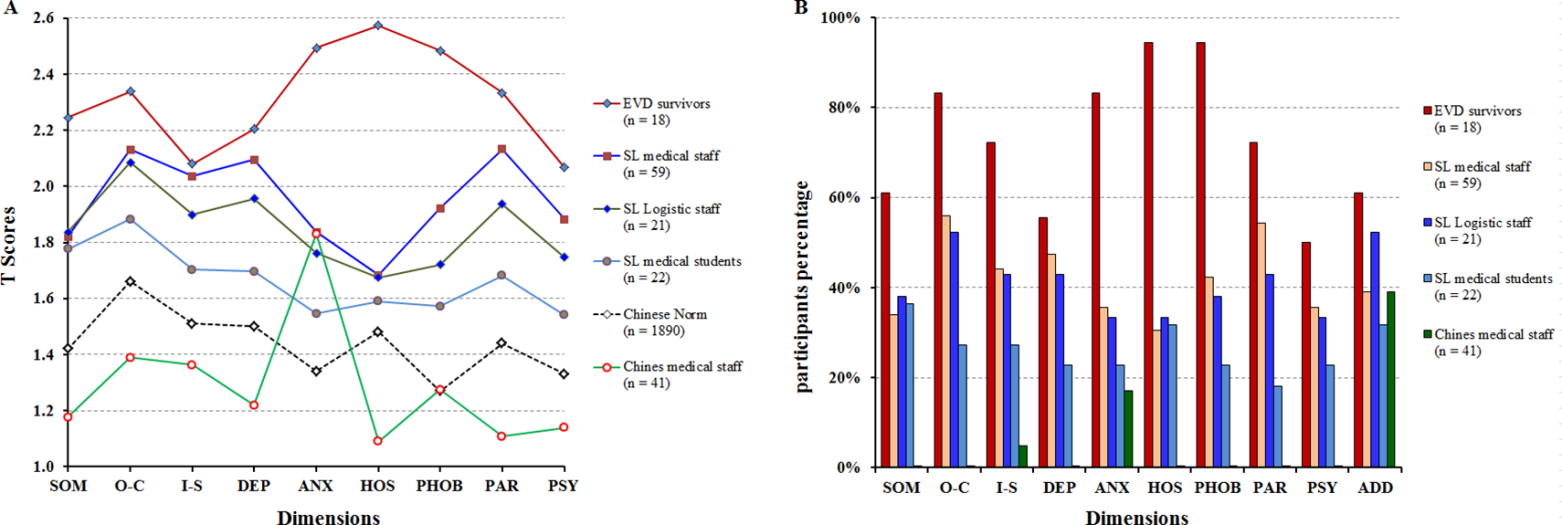
SCL-90-R clinical profiles of EVD survivors and the healthcare workers during the 2014-2015 Ebola Outbreak in Sierra Leone (**A**) The T scores distributions on dimensions of 161 participants. (**B**) The percents of participants with T score more than 2 by 10 dimensions. Note: SOM, somaticization; O-C, obsession-compulsion; I-S, interpersonal sensitivity; DEP, depression; ANX, anxiety; HOS, hostility; PHOB, phobic anxiety; PAR, paranoid ideation; PSY, psychoticism; ADD, additional items; SD, standard deviation. Total sample size across all categories was 161 and for Chinese norms was 1,890.

The mean of General Severity Index (GSI) in EVD survivors, SL medical staff, SL logistic staff, SL medical students, and Chinese medical staff were 2.31 ± 0.57, 1.92 ± 0.62, 1.88 ± 0.68, 1.68 ± 0.73, and 1.25 ± 0.23; Positive Symptom Total (PST) were 62.00 ± 18.93, 43.83 ± 22.87, 38.43 ± 24.25, 34.95 ± 28.10, and 16.76 ± 10.79; Positive Symptom Distress Index (PSDI) were 3.43 ± 0.47, 5.07 ± 2.64, 6.85 ± 5.47, 7.79 ± 7.00, and 11.85 ± 6.79, respectively (Supplementary Table S1). The total GSI and all 10 dimensions of psychological statuses, total PST and total PSDI were the highest in EVD survivors and the lowest in Chinese medical staff (except anxiety and additional items) with significant differences (*P* < 0.05) (Figure 1A). There were no significant differences between SL medical staff and SL logistic staff, SL logistic staff and SL medical students on total GSI, PST and PSDI scores (*P* > 0.05) (Supplementary Table S2).

There were 5 dimensions (obsession-compulsion, anxiety, hostility, phobic anxiety, and paranoid ideation) extremely high (T score > 2.3) in EVD survivors, the proportions of positive symptom number on these 5 dimensions was 83.3%, 83.3%, 94.4%, 94.4%, and 72.2%, respectively. There were 2 dimensions (anxiety and additional items) extremely high (T score > 1.8) in Chinese medical staff with 17.7% and 39.0% of the proportions of positive symptom number (Figure 1B).

### Impact of education level on psychological statues

**Figure 2:**
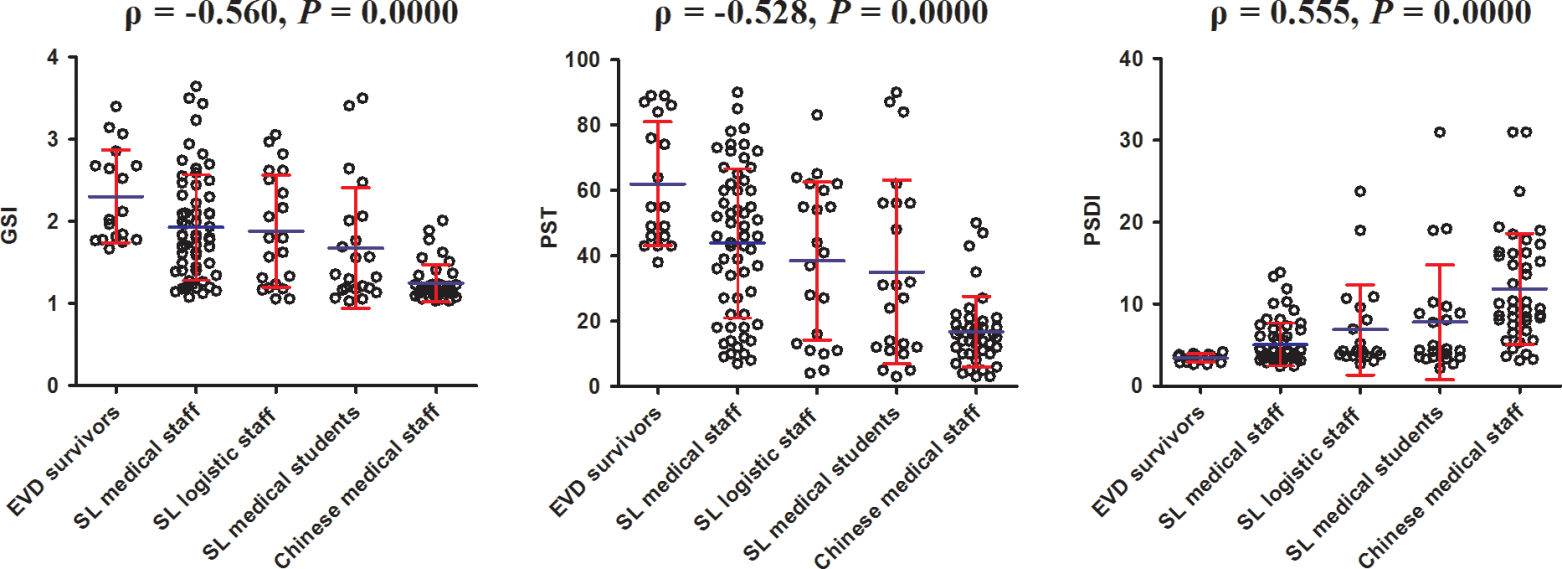
The correlation between GSI,PST, PSDI scores and the education levels of participants Note: GSI, general severity index; PST, the positive symptom yotal ; PSDI, positive symptom distress index

Column scatter plot by groups were used to visualize the relationship between GSI, PST, PSDI and education level, and bivariate Spearman correlation analyses showed that GSI (ρ = −0.560, *P* = 0.0000) and PST (ρ = −0.528, *P* = 0.0000) were associated with university education negatively and PSDI (ρ = 0.555, *P* = 0.0000) related to university education positively (Figure 2).

### Impact of situation on psychological statues

In order to figure out the dynamic transformation of Chinese medical staff mental statues, we conducted the survey on them twice, first time was within 1 week after arriving at Freetown on February 13, 2015, and second time was within 1 week before withdrawing on March 19, 2015. The results showed that the mean of GSI after arriving and before withdrawing were 1.25 ± 0.23 and 1.19 ± 0.23, respectively, and there was no significant difference between them. In detail, the dimensions of anxiety and additional items scores of arriving were higher, and somaticization, obsession-compulsion, depression, hostility, paranoid ideation and psychoticism scores were lower significantly compared with Chinese Norm (*P* < 0.05). Whereas after 2-month working, the scores of anxiety and additional items declined significantly (*P* < 0.05) (Figure 4 and Supplementary Table S3).

**Figure 3:**
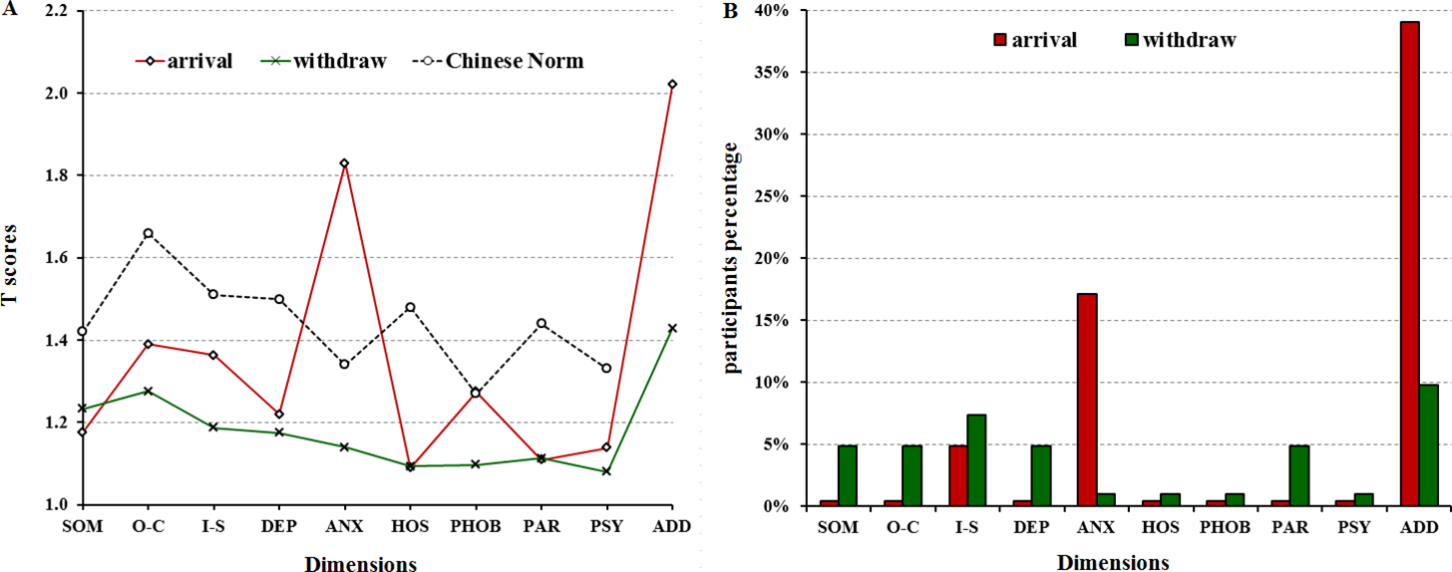
SCL-90-R clinical profiles of Chinese medical staff during the 2014–2015 Ebola Outbreak in Sierra Leone (**A**) The T scores distributions on dimensions of 41 Chinese medical staff. (**B**) The percents of participants with T score more than 2 by 10 dimensions. Note: SOM, somaticization; O-C, obsession-compulsion; I-S, interpersonal sensitivity; DEP, depression; ANX, anxiety; HOS, hostility; PHOB, phobic anxiety; PAR, paranoid ideation; PSY, psychoticism, SD, standard deviation.

**Figure 4:**
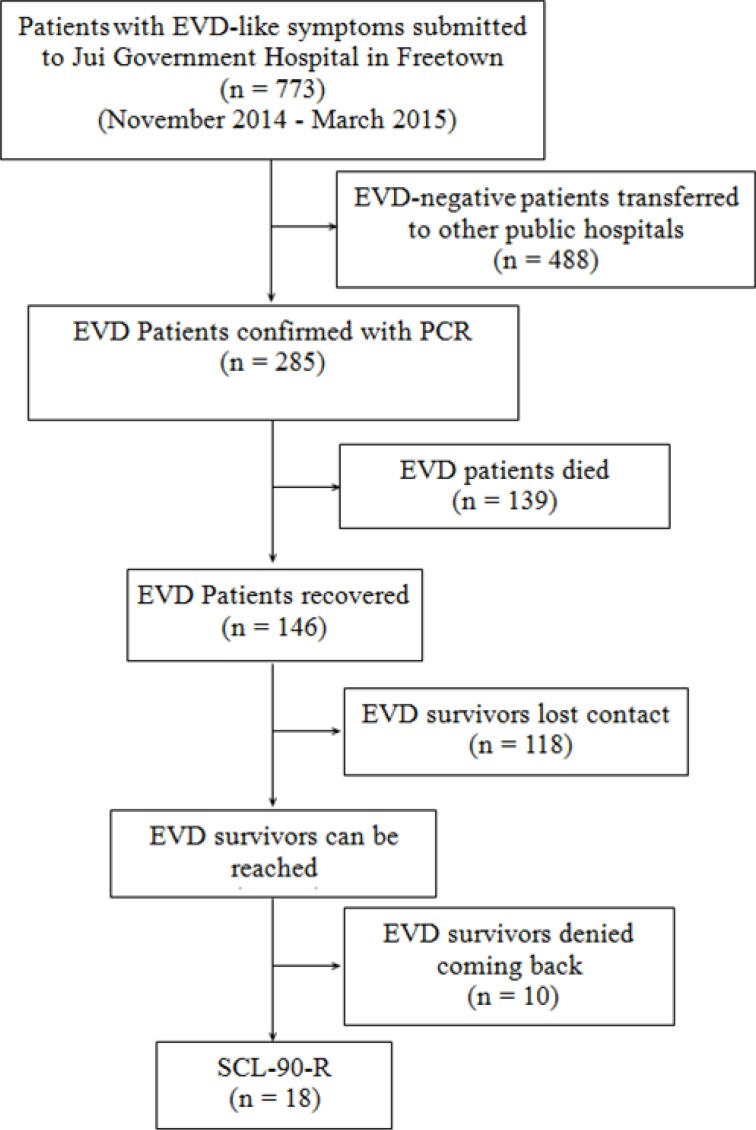
The flow chart of SCL-90-R data collection

## DISCUSSION

The 2014–2015 EVD epidemic was considered to be the largest and most complex outbreak since Ebola has been discovered, affecting multiple countries in West Africa. As to March, 2016, there were 28,616 reported EVD infections (confirmed, probable, and suspected) and 11,310 reported deaths in Guinea, Liberia, and Sierra Leone. The epidemic disease can cause a mental health crisis, the WHO has emphasized on the psychological care of the survivors in its reports, there is much work to be done to ensure that psychological care is a priority in the Ebola response [14–18]. To our knowledge, there is only a small amount of scientific literature available related to this health issue during this Ebola outbreak, so it is necessary to carry out extensive research to assess the psychological impacts of the Ebola. Our study is to evaluate the psychological symptoms in various personal including EVD survivors, logistic staff, and clinical staff, etc., in Sierra Leone.

Our results showed that EVD survivors had extreme somatization, obsession-compulsion, depression, anxiety, hostility, phobic anxiety, paranoid ideation, bad sleep and appetite (defined as T-score higher than 2.2), and highest GSI score. These survivors have been deeply affected by the sufferings and the deaths of their family members and friends, the schools, markets were mostly closed and economic security was also collapsed. Because EVD was featured with high mortality rate, high possibility of being infected, and the strict biosecurity restriction, it produced specific sources of mental stress, which not only prevented survivors from returning to their previous lives and social circles, but also caused the spread of the infection through patient falsification [19–23]. A previous study described mental statues of EVD survivors in Democratic Republic of Congo, which included fear of falling seriously ill, fear of being accused by their neighbors and shame [24]. All of the results of related studies suggested that it is important to formulate the appropriate epidemic response and recovery including plans for addressing the mental health crisis. After control of the outbreak, mental health services should be prepared to manage the substantial increase in need for psychological support. In some cases this can be an opportunity to build back better; a common finding that emergencies present opportunity [25]. The mental health and psychosocial response should be assessed as part of the overall review of the response. A mental health professional should be part of the team reviewing the emergency preparedness plan post outbreak. This integration occurred in Nigeria, and as a result the plan is more comprehensive than would otherwise have been the case.

Our results showed that, in terms of healthcare workers of Sierra Leone, the GSI of SL medical staff, SL logistic staff, SL medical students declined gradually, and SL medical staff, who directly contacted with EVD patients, including nurses, red zone cleaners, and blood team members, had more obvious psychological symptoms, such as obsession-compulsion, interpersonal sensitivity, depression, and paranoid ideation (defined as T-score higher than 2.0). This result is comparable to the previous studies. A research showed that there were about 10% of subject hospital employees in 2003 outbreak of sever acute repiratory syndrome (SARS) had experienced high levels of stress [26]. Another study reported life-events and occupational stress were negative associated with anxiety and psychological distress [27]. The possible reasons include that healthcare workers had to cope with the deaths of colleagues, threats to their lives, and working excessive hours in addition to their own anxiety and fear of contamination, the breakdown of social support systems also increased psychological distress among them [28, 29]. It implied that the potential psychological fallout might arise for medical staff working in the high risk environment, and the emergency response plan should have a list of mental health professionals that have the skills necessary to participate in the response to EID outbreaks, such as EVD.

Our results indicated that the higher level of education received, the less psychological symptoms developed. GSI and PST were associated with university education negatively and PSDI related to university education positively. So in order to control the rumors and false beliefs, and to ease the psychological burdens, the efforts should be made to spread the proper knowledge about the disease in the communities, the role of EVD survivors, governments, volunteers, community heads are important in mitigation the effects [30].

Our results showed Chinese medical team staff had lowest GSI and PST, even lower than Chinese Norm significantly, suggesting that Chinese medical team staff had the best psychological diathesis. In order to fulfill the task successfully, the leaders of Chinese medical team had adopted several measurements before departure, first is necessary medical training, including the information about EVD outbreak, the prevention and treatment procedure for EVD, and situational training programs, etc. Second is physical exercises to enhance the physical fitness, the last is psychological support service to release team members’ pressure and adjust the mental status. In addition, the training programs actually had enhanced supportive relationship among team members. The result from military studies showed that that good relatiohship between colleagues or between leaders and team members are highly associated with mental health [31, 32].

### Limitations

(1) the sample size of EVD survivors was small, because that the management of EVD survivors was somehow incomplete, after they transferred to other ETU, we lost their contact, also some patients concealed their true personal information, even their name, because of stigma. (2) We hadn't collection the dynamic informations from various personnel, the reason was we thought the outbreak of EVD was still last when we withdrew in March 2015, and there might not change much in the psychological status of African people. (3) We hadn't find out the SCL-90-R norms for the Sierra Leone population in literature. However, based on some reports, there was no difference on SCL-90-R dimensions by race, so we used Chinese Norm as an alternative.

## MATERIALS AND METHODS

### Study design and participants

This descriptive cross sectional study was conducted on 143 healthcare associated personnel and 18 EVD survivors. All the enrolled participants were willing to complete the study questionnaire, and without mental disorders, and were able to understand, speak, and write English so as to complete the entire evaluation without difficulties. For those who don't speak English, we provided local interpreters to help them fill out the paper works. The results of the questionnaire were assessed by psychological consultant of Chinese medical team. The consent form and all study procedures were approved by Medical Ethics Committee of the 302 military hospital of China (approve number: 2015181D). Written informed consents were obtained to all subjects before the study.

We investigated 121 healthcare workers at ETC of Sierra Leone-China Friendship Hospital (Jui hospital) at Freetown, they comprised 41 Chinese medical staff who arrived at Freetown, Sierra Leone (SL) on February 13, 2015, and withdrew on March 19, 2015; fifty-nine SL medical staff including nurses and red zone cleaners; twenty-one SL logistic staff including administrators, data collectors and cookers.

Moreover, we also investigated 22 SL medical students studying at Connaught hospital in Freetown, which is the principal adult referral hospital, providing medical and surgical services, medical training and research.

We analyzed the main data of patients’ information who were submitted and treated at our ETC from November 2014 to March 2015 during the 302 military hospital medical team working there, the process of EVD case screening was proformed as previously described [33]. The inclusion criteria of our study was the patients with confirmed EVD by PCR detection, and were cured in our ETC. The exclusion criteria was patients with malaria or infected with other virus, such as yellow fever virus, Lassa fever virus, and HIV. Plasma from all EVD-suspected patients was tested for the glycoprotein gene of Ebola virus subtype Zaire by AccuPower^®^ EBOV Real-Time RT-PCR Kit (Bioneer Corporation, Daejeon, Republic of Korea). All blood samples were treated by Chinese CDC laboratorian according to “Ebola hemorrhagic fever laboratory testing program”. by quantitative reverse transcription PCR [34–36]. There were 773 patients with EVD-like symptoms submitted to our ETC, altogether 488 EVD-negative patients were transferred to other public hospitals, and among 285 EVD patients, there were 139 patients died, and 118 EVD survivors lost contact after discharged, plus 10 survivors denied coming back to Jui hospital because of various excuses, so finally we investigated 18 survivors who were willing to come back (Figure 4).

### Participants’ evaluations

Demographic characteristics and psychological health status data were collected from the participants. The demographic information included age, gender, level of education and profession.

The Symptoms Checklist 90-items, Revised (SCL-90-R), which is a multidimensional self-report symptom inventory, was used to assess psychological status of participants, because it has become one of the most commonly used measures of psychological assessment in medical, clinical and research settings since its development [37]. The inventory is comoposed of 90 items, including somatization (distress arising from perceptions of bodily dysfunctions), obsessive-compulsion (irresistible thoughts, behaviors, or impulses), interpersonal Sensitivity (feelings of personal inadequacy or uneasiness), depression (dysphoric mood and other symptoms associated with depression), anxiety (a tendency toward anxiety as manifest by nervousness, tension, and trembling), hostility (thoughts, feelings, or actions that are associated with a state of anger), phobic anxiety (a persistent fear response to a specific person, place, object, or situation), paranoia (suspiciousness or the fear of loss of autonomy), psychosis (the perception of unusual experiences or interpersonal isolation), and additional items (sleep and appetite) [38]. Each symptom is rated on a five-point scale (1 = normal, 2 = mild, 3 = moderate, 4 = severe, and 5 = extremely) indicating how frequently the participant has experienced these symptoms in the last week. The psychological interviews take about 30–45 minutes in a private environment. Studies have shown that the SCL-90-R has good internal consistency, validity, and test–retest reliability [39].

### Principles and values

The SCL-90-R also has 3 global indices of distress (Global Severity Index, Positive Symptom Total, and Positive Symptom Distress Index), which measure the overall psychological distress level, the intensity of symptoms, and the number of self-reported symptoms. General Severity Index (GSI) is calculated using the sums of the all items divided by 90; the Positive Symptom Total (PST) is a count of all the items with more than 1 score and reveals the number of symptoms that the respondent reports experiencing; Positive Symptom Distress Index (PSDI) is the sum of the values of the items with more than 1 score divided by the PST. Higher score represents worse psychological status.

### Statistical analysis

Descriptive analyses were carried out to explore the data; continuous variables were expressed as mean ± standard deviation and categorical variables as absolute figures and percentages. Chi-squared test was used for categorical variables and Fisher's exact test when appropriate. Continuous variables with norm/skewed distribution were analyzed using students’ *t*-Test/Mann-Whitney U test. Correlation analysis was performed with Spearman rank correlation. A *P* ≤ 0.05 was considered significant. Analysis of all data was performed using GraphPad Prism 5.0 (GraphPad Software, CA, USA) and the SPSS 19.0 for windows (SPSS Inc., IL, USA)

## CONCLUSIONS

In conclusion, the fact that this EVD outbreak will not be the last outbreak of EID is obvious, and a comprehensive emergency response plan must be developed for the future. Not only the medical facilities should be prepared, but also the psychological support for the infected patients, their family members and healthcare workers directly or indirectly involved, should be sustained. Otherwise, all the efforts to control the deadly disease will although control the disease, but will created a new set of patients with social, behavioural, psychological issues.

## SUPPLEMENTARY MATERIALS FIGURES AND TABLES



## Abbreviations

EVD: Ebola virus disease
EID: emerging infectious disease
SCL-90-R: Symptom Checklist-90-Revised
SL: Sierra Leone
GSI: global severity index
PST: positive symptom total
PSDI: positive symptom distress index
SD: standard deviation
CI: confidence interval
