# Supplementary material for: Prevalence of psychological symptoms among Ebola survivors and healthcare workers during the 2014-2015 Ebola outbreak in Sierra Leone: a cross-sectional study
Source: Oncotarget. 2017 Jan 10;8(8):12784–91. doi: 10.18632/oncotarget.14498 (PMC5355054; doi:10.18632/oncotarget.14498)
Supplement: Supplementary file 1 [file oncotarget-08-12784-s001.pdf]

Prevalence of psychological symptoms among Ebola survivors and healthcare workers during the 2014–2015 Ebola outbreak in Sierra Leone: a cross-sectional study

Supplementary Materials

Supplementary Table S1: T scores on the dimensions on the SCL-90-R for current samples and general population (Means ± S.D.)

Dimensions	EVD survivors (*n* = 18)	SL medical staff (*n* = 59)	SL Logistic staff (*n* = 21)	SL medical students (*n* = 22)	Chinese medical staff (*n* = 41)	Chinese Norm (*n* = 1890)
SOM	2.25 ± 0.79	1.82 ± 0.77	1.84 ± 0.74	1.78 ± 0.76	1.18 ± 0.18	1.42 ± 0.44
O-C	2.34 ± 0.52	2.13 ± 0.80	2.09 ± 0.74	1.88 ± 0.49	1.39 ± 0.36	1.66 ± 0.52
I-S	2.22 ± 0.53	2.04 ± 0.80	1.90 ± 0.70	1.70 ± 0.42	1.36 ± 0.33	1.51 ± 0.49
DEP	2.21 ± 0.74	2.10 ± 0.78	1.96 ± 0.90	1.70 ± 0.77	1.22 ± 0.31	1.50 ± 0.47
ANX	2.49 ± 0.59	1.84 ± 0.89	1.76 ± 0.75	1.55 ± 0.77	1.83 ± 0.23	1.34 ± 0.39
HOS	2.57 ± 0.58	1.68 ± 0.71	1.67 ± 0.69	1.59 ± 0.67	1.09 ± 0.13	1.48 ± 0.47
PHOB	2.48 ± 0.57	1.92 ± 0.84	1.72 ± 0.73	1.57 ± 0.30	1.27 ± 0.19	1.27 ± 0.40
PAR	2.33 ± 0.59	2.13 ± 0.84	1.94 ± 0.91	1.68 ± 0.52	1.11 ± 0.19	1.44 ± 0.47
PSY	2.07 ± 0.88	1.88 ± 0.75	1.75 ± 0.78	1.54 ± 0.35	1.14 ± 0.24	1.33 ± 0.39
ADD	2.29 ± 0.83	2.06 ± 0.84	2.14 ± 0.80	1.70 ± 0.30	2.02 ± 0.51	—
GSI	2.30 ± 0.57	1.92 ± 0.62	1.88 ± 0.68	1.68 ± 0.73	1.25 ± 0.23	—
PST	62.00 ± 18.93	43.83 ± 22.87	38.43 ± 24.25	34.95 ± 28.10	16.76 ± 10.79	—
PSDI	3.43 ± 0.47	5.07 ± 2.64	6.85 ± 5.47	7.79 ± 7.00	11.85 ± 6.79	—

Supplementary Table S2: GSI, PST, and PSDI differences among different groups

	EVD survivors (*n* = 18)	SL medical staff (*n* = 59)	SL Logistic staff (*n* = 21)	SL medical students (*n* = 22)
**GSI**				
SL medical staff	**0.0232**			
SL Logistic staff	**0.0455**	0.8051		
SL medical students	**0.0001**	0.1440	0.3586	
Chinese medical staff	**0.0000**	**0.0000**	**0.0000**	**0.0009**
**PST**				
SL medical staff	**0.0031**			
SL Logistic staff	**0.0004**	0.1154		
SL medical students	**0.0000**	0.1486	0.6667	
Chinese medical staff	**0.0000**	**0.0000**	**0.0000**	**0.0005**
**PSDI**				
SL medical staff	**0.0109**			
SL Logistic staff	**0.0121**	0.0543		
SL medical students	**0.0005**	0.0125	0.6274	
Chinese medical staff	**0.0000**	**0.0000**	**0.0049**	**0.0289**

Supplementary Table S3: The dynmic change of psychological dimensions of SCL-90-R among the Chinese medical staff

	Group 1 (Arrival) (*n* = 41)	Group 2 (withdraw) (*n* = 41)	Group 3 (Chinese Norm) (*n* = 1890)	*P* value		
				**1 vs 3**	**2 vs 3**	**1 vs 2**
SOM	1.18 ± 0.18	1.23 ± 0.36	1.42 ± 0.44	0.0005	0.0061	0.4287
O-C	1.39 ± 0.36	1.28 ± 0.31	1.66 ± 0.52	0.0010	0.0000	0.1421
I-S	1.36 ± 0.33	1.19 ± 0.30	1.51 ± 0.49	0.0513	0.0000	0.0169
DEP	1.22 ± 0.31	1.18 ± 0.29	1.50 ± 0.47	0.0002	0. 0000	0.5480
ANX	1.83 ± 0.23	1.14 ± 0.23	1.34 ± 0.39	0.0000	0.0011	0.0000
HOS	1.09 ±0.13	1.09 ± 0.18	1.48 ± 0.47	0.0000	0.0000	1.0000
PHOB	1.27 ± 0.19	1.10 ± 0.20	1.27 ± 0.40	1.0000	0.0067	0.0002
PAR	1.11 ± 0.19	1.11 ± 0.24	1.44 ± 0.47	0.0000	0.0000	1.0000
PSY	1.14 ± 0.24	1.08 ± 0.14	1.33 ± 0.39	0.0019	0.0000	0.1706
ADD	2.02 ± 0.51	1.43 ± 0.49	–	–	–	0.0000
GSI	1.25 ± 0.23	1.19 ± 0.23	–	–	–	0.2410
